# Uncommon neuromyopathy toxicity induced by amiodarone therapy: case report

**DOI:** 10.1186/s43044-025-00631-5

**Published:** 2025-03-19

**Authors:** L. El Bahri, A. Bensied, M. Nessnassi, I. Fellat, Mohamed Cherti

**Affiliations:** Mohammed V University, Rabat, Morocco

**Keywords:** Unusual, Case report, Amiodarone therapy, Neuromyopathy, Toxicity

## Abstract

**Background:**

Amiodarone is an antiarrhythmic drug known for its potential side effects, one of which is neuromyopathy, though it remains relatively rare. This condition can present with muscle weakness, pain, and tremors, potentially leading to functional impairment. The exact mechanisms underlying amiodarone-induced neuromyopathy are not fully understood but may involve both direct muscle toxicity and effects on nerve conduction.

**Case presentation:**

We present the case of a 68-year-old man with symptomatic arrhythmogenic right ventricular dysplasia, receiving long-term amiodarone, experiencing bilateral leg pain and weakness associated with amiodarone use. On clinical examination, motor strength in the lower limbs was rated at 2/5, with decreased tactile sensation. The biological assessment showed normal level of creatine kinase and C-reactive protein. The spinal MRI was normal. Electromyography “EMG” revealed a non-length dependent sensorimotor demyelinating polyneuropathy. After discontinuing amiodarone, both mobility and function showed significant improvement.

**Conclusion:**

These observations highlight the importance of performing neurologic examinations in patients treated with amiodarone to identify even rare complications, such as neuromyopathy. Importantly, neuromyopathy is often reversible following discontinuation of the drug.

## Background

Amiodarone is an iodine-rich antiarrhythmic medication commonly used to manage various cardiac rhythm abnormalities. Despite its efficacy, amiodarone is associated with a range of potential side effects affecting multiple organ systems. While these side effects are typically mild, 10–15% of patients may need to discontinue the medication due to toxicity [1]. The most common adverse effects associated with long-term use include thyroid dysfunction, corneal microdeposits, and pulmonary and hepatic toxicity [2]. Peripheral neuropathy is another known complication, affecting approximately 10% of patients on amiodarone therapy [3].

Although neuromyopathy is a rare complication of amiodarone, it should be considered in patients presenting with relevant symptoms. Here, we present a case of amiodarone-induced neuropathy following two years of treatment with 400 mg of oral amiodarone.

## Case presentation

A 68-year-old male presented to our institution with a chief complaint of subacute bilateral leg pain and weakness, resulting in complete loss of independent ambulation. His medical history included arrhythmogenic right ventricular dysplasia complicated with ventricular tachycardia, which required the implantation of an ICD in 2009, with device replacement in 2019. He also had a history of infectious endocarditis due to *Staphylococcus aureus*, treated with ICD extraction and initiation of 400 mg of amiodarone therapy for rate control in 2021.

Two years after the initiation of amiodarone therapy, the patient experienced a transient episode of tremor, which was initially overlooked. This was followed by the development of bilateral leg pain and weakness, progressively leading to functional impairment. The pain exhibited a migratory pattern but remained localized to the lower limbs, without any associated bowel or bladder dysfunction.

On physical examination, segmental muscle strength in the lower limbs was 2/5, with 3/5 strength in ankle dorsiflexion. Osteotendinous reflexes were absent bilaterally, and tactile hypoesthesia extended up to the knees.

The biological assessment revealed normal levels of creatine kinase and C-reactive protein. Additionally, vitamin B12 levels were within the reference range, and viral serologies for hepatitis B (HBV), hepatitis C (HCV), HIV, syphilis, Epstein–Barr virus (EBV), and cytomegalovirus (CMV) were negative. Thyroid-stimulating hormone (TSH), calcium, magnesium, and liver function tests were all within normal limits.

Spinal MRI findings were unremarkable.

Electromyography (EMG) demonstrated a non-length-dependent sensorimotor demyelinating polyneuropathy, aligning with the clinical presentation of acute polyradiculoneuritis [Table 1]. Sural nerve biopsy was not performed due to financial constraints.

**Table 1:** Motor nerve conduction studies (MNC)

Nerf/Sites	Muscle	Latency ms	Amplitude mV	Duration ms	Rel Amp %	Segments	Distance mm	Lat Diff ms	Velocity m/s	Vel CT m/s
Distal tibial nerve (D tibial)–fibular common peroneal nerve (FGO)										
Ankle	FGO	9.27	2.2	7.45	100	Ankle–FGO	60			
Popliteal fossa	FGO	20.47	1.3	8.80	61.2	Popliteal fossa–ankle	405	11.20	36	36.2
G Tibial–FGO										
Ankle	FGO	8.54	2.5	7.29	100	Ankle–FGO	60			
Popliteal fossa	FGO	19.79	1.8	8.18	70.8	Popliteal fossa–ankle	400	11.25	36	35.6
Median nerve and its compound action potential (distal Median-CAP)										
Wrist	Abductor pollicis brevis (APB)	4.27	4.9	6.61	100	Wrist–APB	70			
Elbow	APB	9.64	4.3	6.88	87.4	Elbow–wrist	260	5.36	48	48.4
Upper arm	APB	11.93	4.4	7.14	104	upper arm–elbow	110	2.29	48	48
Proximal Median-CAP										
Wrist	APB	4.53	6.3	8.28	100	Wrist–APB	70			
Elbow	Abductor pollicis group (APG)	10.10	4.3	6.82	68.6	Elbow–wrist	270	5.57	48	48.4
Upper arm	APG	12.14	3.9	9.48	91.1	Upper arm–elbow	100	2.03	49	49.2
Distal cubital tunnel (D cubital)–abductor digiti minimi (ADM)										
Wrist	Abductor digiti minimi (ADM)	5.42	5.4	8.39	100	Wrist -ADM	70			
B elbow	ADM	10	4.2	8.91	77.5	B elbow–wrist	220	4.58	45	45
A elbow	ADM	13.18	3.8	8.96	90.3	A elbow–B elbow	120	3.18	38	37.8
Axilla	ADM	14.64	3.5	9.32	91.9	Axilla–A elbow	95	1.46	65	65.1
						A elbow–wrist		7.76		
Proximal cubital tunnel (G cubital)–abductor digiti minimi (ADM)										
Wrist	ADM	4.48	6.1	7.97	100	Wrist -ADM	70			
B elbow	ADM	10.21	4.2	8.28	69.1	B elbow–wrist	250	5.73	44	43.6
A elbow	ADM	14.38	3.9	8.70	91.9	A elbow–elbow	175	4.17	42	42
						A elbow–wrist		9.90		
Distal peroneal nerve (D peroneal)–extensor digitorum brevis (EDB)										
Ankle	Peroneus	7.71	1	5.73	100	Ankle peroneus	80			
Fibular head	Peroneus	17.92	0.8	8.33	79.6	Fibular head ankle	320	10.21	31	31.3
Popliteal fossa	Peroneus	21.35	1	6.41	127	Popliteal fossa–fibular head	100	3.44	29	29.1
						Popliteal fossa–ankle		13.65		
Proximal peroneal nerve (G peroneal)–extensor digitorum brevis (EDB)										
Ankle	Peroneus	6.04	0.7	5.42	100	Ankle peroneus	80			
Fibular head	Peroneus	19.95	0.6	5.42	83	Fibular head ankle	315	13.91	23	22.7
Popliteal fossa	Peroneus	23.28	0.6	5.57	104	Popliteal fossa–fibular head	85	3.33	26	25.5
						Popliteal fossa–ankle		17.24		
Distal peroneal (D peroneal)–tibialis anterior										
Fibular head	Tibialis anterior	5.26	2.5	9.90	100	Fibular head–Tib Ant				
Popliteal fossa	Tibialis anterior	6.56	2.5	10.52	100	Popliteal fossa–fibular head	65	1.30	50	49.9
Proximal peroneal (G peroneal)–tibialis anterior										
Fibular head	Tibialis anterior	9.13	1.8	11.15	100	Fibular head–tibialis anterior				
Popliteal fossa	Tibialis anterior	10.10	1.9	10.68	105	Pop fossa–fibular head	85	1.98	43	42.9
G axillary-deltoid										
Supra-clavicular fossa	Deltoid	7.71	2.0	10.68	100					

Additionally, the electrocardiogram (EKG) demonstrated atrial fibrillation with a heart rate of 90 bpm and a left anterior hemiblock.

Upon admission, amiodarone was discontinued, and rate control was initiated with a beta-blocker. With physical therapy and active rehabilitation, the patient gradually regained function in the lower extremities. After nearly one month of hospitalization, motor strength in the lower limbs improved to 4/5, and the patient was able to ambulate with crutches.

At the three-month follow-up, the patient had regained his baseline functional status prior to amiodarone treatment. Additionally, he had not experienced any episodes of palpitations or syncope since discharge.

## Discussion

Amiodarone is an antiarrhythmic drug known for its wide range of potential side effects. As an amphiphilic compound, it forms intralysosomal lipid complexes in various tissues, contributing to pathological changes and toxicity [4].

Neuromyopathy, although rare, is a recognized adverse effect of amiodarone therapy. It is characterized by significant proximal and distal muscle weakness, loss of distal sensation, and diminished muscle stretch reflexes [5], with the lower extremities typically more affected than the upper limbs. The onset of neuromyopathy is highly variable, with symptoms emerging from a few months to several years after treatment initiation [6]. This variability complicates diagnosis and highlights the importance of early recognition, particularly in patients presenting with unexplained neuromuscular symptoms.

A distinctive feature of our case was the normal creatine kinase (CK) levels. Although CK elevation is commonly used as a biomarker for muscle injury and is often associated with myopathic processes, its absence does not rule out neuromuscular pathology. In particular, amiodarone-induced neuromyopathy can present with significant muscle weakness and sensory deficits while maintaining normal CK levels. This paradox is thought to result from the primarily neurogenic component of amiodarone toxicity, where muscle degeneration occurs in the absence of overt muscle fiber necrosis or inflammatory myositis, leading to minimal CK leakage into the bloodstream [7].

Consequently, relying solely on CK levels for the diagnosis of amiodarone-induced myopathy may result in underdiagnosis or delayed recognition of the condition. Previous reports have shown that CK levels can remain within normal limits even in patients with profound neuromuscular dysfunction, highlighting the limitations of CK as a diagnostic tool in drug-induced myopathies. Therefore, a comprehensive clinical evaluation is essential, incorporating electrophysiological studies such as electromyography (EMG) and nerve conduction studies (NCS) to assess the extent of neuromuscular involvement. EMG findings in amiodarone-induced toxicity typically reveal features of a demyelinating sensorimotor polyneuropathy, often with a length-independent pattern affecting both proximal and distal muscles.

Generally, muscle strength improves gradually after the discontinuation of amiodarone, with full recovery often taking up to six months [8]. In our case, the patient’s muscle weakness showed improvement within three weeks, and ambulation was regained three months following amiodarone cessation.

Although a sural nerve biopsy was not performed due to logistical constraints, we strongly suspect amiodarone-induced neuropathy based on several key factors. First, the patient’s clinical presentation, marked by progressive muscle weakness predominantly in the lower limbs, aligns with typical symptoms of drug-induced neuropathy. Furthermore, the patient’s motor function showed significant improvement following the discontinuation of amiodarone, further supporting the likelihood of amiodarone-induced toxicity. Second, the electromyographic findings revealed a mixed sensorimotor polyneuropathy, which is consistent with the known neuropathic complications associated with amiodarone use.

Additionally, the exclusion of other potential causes of neuromyopathy, such as metabolic, infectious, or other pharmacological agents, strengthens our diagnosis. Given the temporal association between amiodarone initiation and symptom onset, coupled with the absence of alternative explanations, we conclude that the neurologic findings in our patient are most likely attributed to amiodarone-induced neuropathy.

This case highlights the importance of considering amiodarone toxicity in patients with unexplained neuromuscular symptoms, especially when typical diagnostic markers, such as creatine kinase, may not be elevated.

## Conclusion

In conclusion, amiodarone-induced neuromyopathy, although uncommon, should be considered in patients presenting with unexplained neuromuscular symptoms, particularly those with a history of prolonged amiodarone use. Early detection and prompt discontinuation of the drug can lead to significant recovery, highlighting the importance of regular monitoring in patients receiving long-term amiodarone therapy.

## Data Availability

No datasets were generated or analyzed during the current study.

## Abbreviations

EKG: Electrocardiogram
EMG: Electromyography
MNC: Motor nerve conduction
MRI: Magnetic resonance imaging
CK: Creatine kinase
ADM: Abductor digiti minimi
EDB: Extensor digitorum brevis

## References

[1] Kang HM (2007) Amiodarone-induced hepatitis and polyneuropathy. Korean J Intern Med 22:225–229. 10.12677/acrvm.2024.121001 17939344 10.3904/kjim.2007.22.3.225 PMC2687697

[2] Podrid PJ (1995) Amiodarone: reevaluation of an old drug. Ann Intern Med 122:689–700. 10.7326/0003-4819-122-9-199505010-00008 7702232 10.7326/0003-4819-122-9-199505010-00008

[3] Martinez-Arizala A, Sobol SM, McCarty GE, Nichols BR, Rakita L (1983) Amiodarone neuropathy. Neurology 33:643–645. 10.1212/WNL.33.5.643 6302557 10.1212/wnl.33.5.643

[4] Shepherd NA, Dawson AM, Crocker PR, Levison DA (1987) Granular cells as a marker of early amiodarone hepatotoxicity: a pathological and analytical study. J Clin Pathol 40:418–423 3584485 10.1136/jcp.40.4.418 PMC1140975

[5] Arnaud A, Neau JP, Rivasseau-Jonveaux T, Marechaud R, Gil R (1992) Neurologic toxicity of amiodarone: 5 case reports. Rev Med Interne 13:419–422 1344923

[6] Pulipaka U, Lacomis D, Omalu B (2002) Amiodarone-Induced neuromyopathy: three cases and a review of the literature. J Clin Neuromuscul Dis 3:97–105. 10.1097/00131402-200203000-00001 19078662 10.1097/00131402-200203000-00001

[7] Jafari-Fesharaki M, Scheinman MM (1998) Adverse effects of amiodarone. Pacing Clin Electro 21:108–120. 10.1111/j.1540-8159.1998.tb01068.x 10.1111/j.1540-8159.1998.tb01068.x 9474655

[8] Stanton MM (2020) Amiodarone-induced neuromyopathy in a geriatric patient. BMJ Case Rep 13:236620–236710. 10.1136/bcr-2020-236620 10.1136/bcr-2020-236620 PMC7643464 33148598

